# Correction: Structural engineering of MXenes for enhanced magnesium ion diffusion: a computational study

**DOI:** 10.1039/d6ra90006b

**Published:** 2026-01-29

**Authors:** Mingxiao Ma, Xiangyu Yao, Jianglong Wang, Xingqiang Shi, Ruining Wang, Ruqian Lian, Dongxiao Kan, Chenyang Jing

**Affiliations:** a Key Laboratory of Optic-Electronic Information and Materials of Hebei Province, Hebei Research Center of the Basic Discipline for Computational Physics, College of Physics Science and Technology, Hebei University Baoding 071002 China 16632719716@163.com rqlian@126.com; b Northwest Institute for Non-ferrous Metal Research Xi’an 710016 P. R. China kandx@c-nin.com

## Abstract

Correction for ‘Structural engineering of MXenes for enhanced magnesium ion diffusion: a computational study’ by Mingxiao Ma *et al.*, *RSC Adv.*, 2025, **15**, 16219–16227, https://doi.org/10.1039/D5RA01985K.

The authors regret that the funding details presented in the Acknowledgements section of the original article are incorrect. The amended text for the Acknowledgements section is presented below.

“This work was supported by the Hebei Natural Science Foundation Youth Foundation (No. B2024201089), the Advanced Talents Incubation Program of the Hebei University (No. 521000981394), the Scientific Research and Innovation Team of Hebei University (Grant No. IT2023B03) and the High-Performance Computing Center of Hebei University.”

The Royal Society of Chemistry apologises for these errors and any consequent inconvenience to authors and readers.

